# Assessing the Spatiotemporal Variation and Impact Factors of Net Primary Productivity in China

**DOI:** 10.1038/srep44415

**Published:** 2017-03-10

**Authors:** Xue Wang, Kun Tan, Baozhang Chen, Peijun Du

**Affiliations:** 1 China University of Mining and Technology, Jiangsu Key Laboratory of Resources and Environment Information Engineering, Xuzhou, 221116, China; 2 Nanjing University of Science and Technology, School of Computer Science and Engineering, Nanjing, 210093, China

## Abstract

In this study, the net primary productivity (NPP) in China from 2001 to 2012 was estimated based on the Carnegie-Ames-Stanford Approach (CASA) model using Moderate Resolution Imaging Spectroradiometer (MODIS) and meteorological datasets, and the accuracy was verified by a ChinaFLUX dataset. It was found that the spatiotemporal variations in NPP present a downward trend with the increase of latitude and longitude. Moreover, the influence of climate change on the evolution of NPP shows that NPP has had different impact factors in different regions and periods over the 12 years. The eastern region has shown the largest increase in gross regional product (GRP) and a significant fluctuation in NPP over the 12 years. Meanwhile, NPP in the eastern and central regions is significantly positively correlated with annual solar radiation, while NPP in these two regions is significantly negatively correlated with the growth rate of GRP. It is concluded that both the development of the economy and climate change have influenced NPP evolution in China. In addition, NPP has shown a steadily rising trend over the 12 years as a result of the great importance attributed to ecological issues when developing the economy.

With the rapid progress of industrialization and urbanization in China, the atmospheric concentrations of greenhouse gases such as carbon dioxide continue to increase because of human activities such as fossil fuel burning, environmental pollution, and land-use change^1^. To reveal the causes of environmental degradation, the carbon cycle in the various terrestrial ecosystems needs to be better understood. As there are many different processes that together comprise the carbon cycle, the indicators of the cycle can be divided into the component fluxes, i.e., gross primary production (GPP) and ecosystem respiration (RE); the net fluxes, i.e., net ecosystem productivity (NEP) and net primary productivity (NPP); and the exchange fluxes, i.e., net ecosystem carbon exchange (NEE). The amount of chemical energy as biomass that the primary producers create is called GPP^2^, which is the first process of the exchange of carbon dioxide that assimilates atmospheric carbon dioxide into the ecosystem^3^. A certain fraction of this chemical fixed energy is used by the primary producers for heterotrophic respiration (RH) and autotrophic respiration (RA), which together comprise respiration (RE)^4^. NEP describes the photosynthetic product by subtracting RE from GPP in an ecosystem, i.e., NEP = GPP−RE. Defined as the amount of organic matter produced by green plants per unit of time and area^5^, NPP is the indicator of the balance between the carbon gained by GPP and the carbon released by plant respiration, which is indicated by RA, i.e., NPP = GPP−RA^2^. NEE is the indicator of equilibrium between photosynthesis and respiration in the ecosystem. A negative sign for NEE, which is the same as NEP numerically, denotes carbon entering the ecosystem from the atmosphere, whereas a positive sign denotes carbon release from the ecosystem into the atmosphere^6^.

As one of the key components of the terrestrial carbon cycle, NPP accounts for most of the carbon flux between the atmosphere and biosphere among the pools and fluxes that make up the cycle^7^. Therefore, accurate retrieval of NPP for the various terrestrial ecosystems is important for ecosystem management and the study of the carbon cycle, and has been the subject of a great deal of attention from academics and governmental agencies^8^.

Zhu *et al*.^9^ pointed out that the three main kinds of models for terrestrial NPP estimation are: (1) climate-productivity relationship models; (2) eco-physiological process models; and (3) light utilization efficiency (LUE) models. Remote sensing is commonly used in the LUE models, which are efficient methods for both regional- and global-scale NPP estimation. Information about vegetation types and/or temperature/water availability is also commonly incorporated into the LUE models^10^. One such technique is the Carnegie-Ames-Stanford Approach (CASA) model for estimating NPP from remote sensing data^11^. CASA is a widely recognized NPP model that downregulates photosynthetic efficiency in response to short-term adverse temperatures or dry soil conditions^12^.

The other approach, which was used in this study, is to calculate the NPP based on the eco-physiological processes using the eddy covariance (EC) technique of microclimatology. In this study, we calculated GPP based on a ChinaFLUX dataset. The ChinaFLUX network is a long-term national network of micrometeorological flux measurement sites which has been operating in China since 2002. The NPP per unit of carbon assimilated by gross photosynthesis (i.e., the carbon-use efficiency, CUE)^13^ is represented by the NPP/GPP ratio, which allows researchers to calculate NPP directly from GPP, or vice versa^14^. The ratio was observed to be approximately constant among diverse vegetation types by Gifford^15^; however, Zhang *et al*.^16^ pointed out that areas with lower CUE values largely consist of wet and warm environments, and their counterparts comprise relatively dry and cold environments. Zhang *et al*.^17^ obtained the mean ratio of the NPP/GPP of different vegetation types using 10 years of global remote sensing data from 2000 to 2009, which we used in this study to calculate the NPP from the observed GPP.

The objectives of this study were as follows: (1) to estimate the NPP of the main territories in China during the period from 2001 to 2012, based on MODIS and meteorological data under the CASA model; (2) to apply the flux data from the ChinaFLUX network to verify the accuracy of the model; (3) to assess the spatiotemporal variation of NPP over the study area and explore the influence of climate change on the evolution of NPP; and 4) to analyze the relationship between the dynamics of NPP and human factors such as GRP and population, alongside economic regionalization.

## Methods

### Data

China, as one of the largest countries in the word, shows a clear latitudinal and longitudinal pattern of ecosystems because of the diverse climatic zones, which have been delineated as plateau, cold (sub-cold) temperate, temperate, warm temperate, subtropical, and tropical climate zones. Geographically, the northwestern part of China is located in the hinterland of the Eurasian continent, the southeastern part of China faces the Pacific, and the Qinghai–Tibet Plateau in the southwest of China is some of the highest terrain on earth^18^. The data time period in this study was from 2001 to 2012, which includes the 10th Five Year Plan and the 11th Five Year Plan, which were a series of social, economic, and ecological development initiatives shaped by China through the plenary sessions of the Central Committee and National Congress.

**Figure 1:**
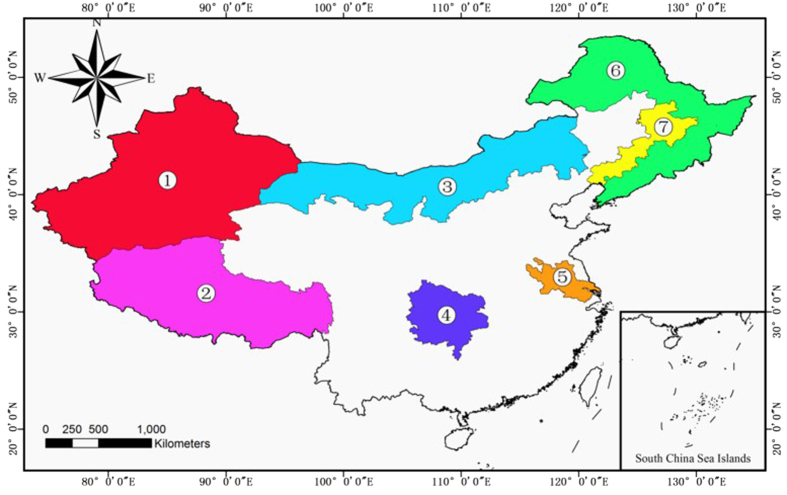
Map of the whole of China and the important geographical areas referenced in this paper. This figure was generated by ArcGIS for Desktop 10.0 (http://desktop.arcgis.com). Area 1 is Xinjiang, the north of area 2 is Kunlun Mountains, area 3 denotes the mainly regions of Inner Mongolia, area 4 is cloudy all the year round whose data is sometimes invalid in MCD12Q1, area 5 is the Huai River basin and the Daxing’an Mountains, the Xiaoxing’an Mountains, and the Changbai mountains are located in area 6, area 7 have Northeast Plain of China and heavy industry areas such as Harbin, Changchun, and Shenyang.

In this study, the data we utilized covered terrestrial China, except for the southwest of Taiwan and the South China Sea Islands. Some important geographical areas that are referenced in the text have been marked in Fig. 1.

Four types of datasets were used in this study: (1) remote sensing data; (2) a meteorological monitoring dataset; (3) a carbon dioxide flux dataset at the ecosystem level; and (4) a vector map of China. Specifically, the datasets were: (1) MODIS and normalized difference vegetation index (NDVI) datasets derived from the extracted data of the Terra satellite at a spatial resolution of 1 × 1 km^2^. The Land Cover Type 4 classification system of MCD12Q1^19^ was used in this study, and all the tiles of the land-cover data were merged together and converted into TIFF format using the MODIS Reprojection Tool (http://lpdaac.usgs.gov/tools/modis_reprojection_tool). The NDVI product, which is a MODIS derivative obtained from the Chinese Geospatial Data Cloud (http://www.gscloud.cn/), has a temporal resolution of one month, and the MODIS land-cover product is a yearly product from NASA (http://modis.gsfc.nasa.gov/). (2) The monthly mean temperature, precipitation, and sunshine duration for the study area were gathered from the China Meteorological Data Sharing Service System (http://cdc.nmic.cn) and spatially interpolated using the kriging interpolation method to obtain the same spatial resolution as the remote sensing images. The first two kinds of data used in this study were georeferenced to the geographic Lat./Lon. projection using the WGS84 datum. (3) The ChinaFLUX dataset^18^ was provided by the ChinaFLUX program, which is a long-term national network of micrometeorological flux measurement sites that measure the net exchange of carbon dioxide, water vapor, and energy between the biosphere and atmosphere^20^. The dataset we used was obtained from eight observation sites: Changbaishan broad-leaved Korean pine mixed forest (CBS), Qianyanzhou subtropical coniferous plantation (QYZ), Dinghushan subtropical evergreen broad-leaved forest (DHS), Xishuangbanna tropical evergreen broadleaf forest (XSBN), Inner Mongolia typical temperate grassland (NM), Haibei alpine meadow (HB), Dangxiong alpine steppe-meadow (DX), and Yuchen warmer temperate dry farming cropland (YC). (4) The Chinese vector map was downloaded from the website of the National Earth System Science Data Sharing Infrastructure of China (http://www.geodata.cn), using the WGS84 geographic coordinate system to produce Fig. 1.

Subtracting NEE (which was directly measured by the EC approach) from RE gives GPP^21^. We utilized the GPP to estimate the observation sites’ NPP by the NPP/GPP ratio.


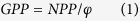


However, the NPP/GPP ratio *φ* shows a considerable spatial variation associated with the ecosystem type, geographical location, and climate^16^. Zhang *et al*.^17^ simulated the NPP/GPP ratio of a variety of ecosystem types. From these results, we chose 0.5853 (the ratio of the evergreen needleleaf forest ecosystem type) as the counterpart of QYZ, 0.4125 (evergreen broadleaf forest) as the counterpart of XSBN, 0.5488 (mixed forest) as the counterpart of DHS, 0.5523 (grass) as the counterpart of NM, HB, and DX, and 0.5399 (crops) as the counterpart of YC, and we obtained the NPP simulation results by month.

### CASA model

Most satellite-based NPP models have been based on the theory of LUE proposed by Monteith^22^, who suggested that plant productivity is strongly related to the intercepted solar radiation, and thus can be estimated as the product of the intercepted solar radiation and its conversion efficiency into plant photosynthesis. The CASA model^23^, which is based on the LUE concept, modified by temperature and moisture stress scalars, was established to calculate monthly terrestrial NPP. The NPP estimation process we utilized introduces both the vegetation types and their classification accuracies. The meteorological dataset was used to estimate both the moisture and temperature stress factors, while the vegetation types were considered when determining the maximum and minimum of the NDVI and when calculating the real LUE^23^.

Theoretically, in the CASA model, NPP is estimated using the absorbed photosynthetically active radiation (APAR) and the real LUE.


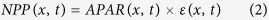


where *NPP(x*, *t*) represents the NPP at month *t* for grid position *x* (unit: *gC* · *m*^−2^ · *month*^−1^), *APAR(x*, *t*) is the APAR at month *t* for grid position *x* (unit: *MJ* · *m*^−2^ · *month*^−1^), and *ε(x*, *t*) is the real LUE (unit: *gC* · *MJ*^−1^)^24^. APAR is determined by both the total solar radiation and the characteristics of the plant canopy, and can be calculated as:


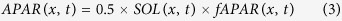


where *SOL* represents the total solar radiation, which can be obtained by establishing the relationship model between the sunshine duration included in the meteorological dataset and the solar radiation for grid position *x* (unit: *MJ* · *m*^−2^ · *month*^−2^)^25^. *fAPAR(x*, *t*) is the fraction of APAR absorbed by the plant canopy, where 0.5 represents the proportion of the radiation which can absorbed by plants (0.38–0.71 μm)^26^. Considering the good linear correlation between *NDVI* and *fAPAR*^10^, *NDVI*, which is calculated from the infrared and the near-infrared channels, is utilized to obtain *fAPAR*.


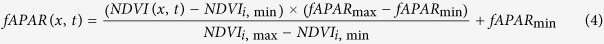


where *NDVI*_*i*,min_ and *NDVI*_*i*,max_ indicate the maximum and minimum of vegetation type *i*, respectively. In this study, we used 95% and 5% in the NDVI histogram as the maximum and minimum of each vegetation type^27^. *fAPAR*_max_ and *fAPAR*_min_ are constants, with values of 0.001 and 0.95, respectively.

### Light utilization efficiency (LUE)

The LUE in realistic conditions is influenced by environmental drivers such as temperature and vapor pressure deficit (VPD), which can retrieved from the meteorological factors.


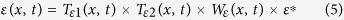


where *T*_ε1_ and *T*_ε2_ represent the effect of high and low temperature on LUE, respectively; *W*_ε_ represents the effect of moisture on LUE; and ε^*^ represents the maximum light utilization rate under ideal conditions. *T*_ε1_ denotes the depressant effect on NPP of high and low temperatures restricting the process of photosynthesis^28^.

The Land Cover Type 4 classification system of the MODIS land-cover product (MCD12Q1) which was used in the CASA model consists of the following categories: water, evergreen needleleaf vegetation, evergreen broadleaf vegetation, deciduous needleleaf vegetation, deciduous broadleaf vegetation, annual broadleaf vegetation, annual grass vegetation, and non-vegetated and urban. The maximum LUE ε^*^ (unit:*gC* · *Mj*^−1^) for each vegetation type was given by Zhu and Pan^29^ as follows: we chose 0.389 as the counterpart of water, 0.985 as the counterpart of evergreen needleleaf vegetation, 0.485 as the counterpart of evergreen broadleaf vegetation, 0.692 as the counterpart of deciduous needleleaf vegetation, HB, and DX, and 0.542 as the counterpart of deciduous broadleaf vegetation, annual broadleaf vegetation, annual grass vegetation, non-vegetated and urban.

### Pearson correlation coefficient

To analyze the correlation between the interannual variation of NPP and the impact factors, we calculated the Pearson correlation coefficient by pixel over the 12 years.


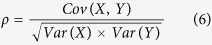


where *Var(X*) and *Var(Y*) represent variables *X* and *Y,* which here mean the annual NPP and the impact factors, respectively. *Cov(X*, *Y*) represents the covariance between the two variables.

## Results and Discussion

### Verification of the results by ChinaFlux data

The ChinaFlux dataset provides the daily GPP of the observation sites from 2003 to 2005, which we used as annual GPP. Using the annual GPP and the NPP/GPP ratio mentioned above, we could obtain the annual NPP, which was used to validate the precision of the result obtained by the CASA model. We located the grid position of the result of the CASA model based on the observing sites’ longitude and latitude, and we then used a 3 × 3 mean filter mask to calculate the average of this grid position and its periphery to represent the model NPP. The comparison between the observed results and the model results in this study includes the eight sites during 2004 and 2005, but only six sites in 2003 (without NM and DX), because of missing observation data.

We calculated the average relative error by:


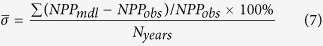


**Figure 2:**
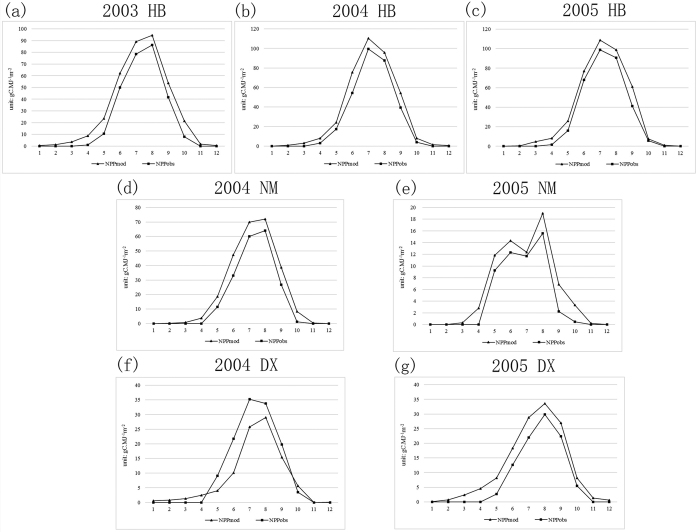
Comparison between the monthly model NPP and observed NPP for the NM, DX, and HB sites.

The average relative errors of eight sites are as follow: the error of CBS is 8.18%, QYH is 3.90%, DHS is 12.02%, XSBN is 5.68%, NM is 34.99%, DX is 31.66% and YC is 12.42%. There are five under 20% but were greater than 20% for the NM, HB, DX sites. The model NPP was greater than the observed NPP for NM and HB, but was smaller for the DX site, whereas the range of the model NPP in all eight sites was in agreement with Zhu and Pan’s estimated results. The reason for this may be that remote sensing images can reflect the evolution of plants over a large scale, but the observation sites covered only a few hectares, so the differences between the two scales seriously affected the average relative error calculation in NM, HB, and DX for typical temperate grassland, alpine meadow, and alpine steppe-meadow, where the average NPP was 124.07 *gc* · *m*^−2^ · *a*^−1^, 329.41 *gc* · *m*^−2^ · *a*^−1^, and 109.03 *gc* · *m*^−2^ · *a*^−1^, respectively. In contrast with the other sites, for NN, HB, and DX, the NPP varied significantly around the three sites, which can be seen in Fig. 2.

We selected the monthly NPP values of NM, DX, and HB as follows:

Figure 2 shows that the model and observed NPP match well by month in the three sites, but the model NPP is smoother. The reason for this could be the use of empirical values for all pixels of the same class, and the kriging interpolation in the meteorological factor calculation, which could act as a smoothing process, ignoring the diversity of the different locations.

The monthly results of the CASA model agree with the ChinaFLUX observation data, but the average relative errors of the three sites are greater than 20%. The reason for this may be that the relative error function (Formula 7), i.e., the denominator for the observation data, which is very small in the HB, NM, and DX sites (about 100–300 *gc* · *m*^−2^ · *a*^−1^, while it is 600–1200 *gc* · *m*^−2^ · *a*^−1^ in the other five sites), has magnified the errors.

### The spatiotemporal variation and impact factors of NPP

**Figure 3:**
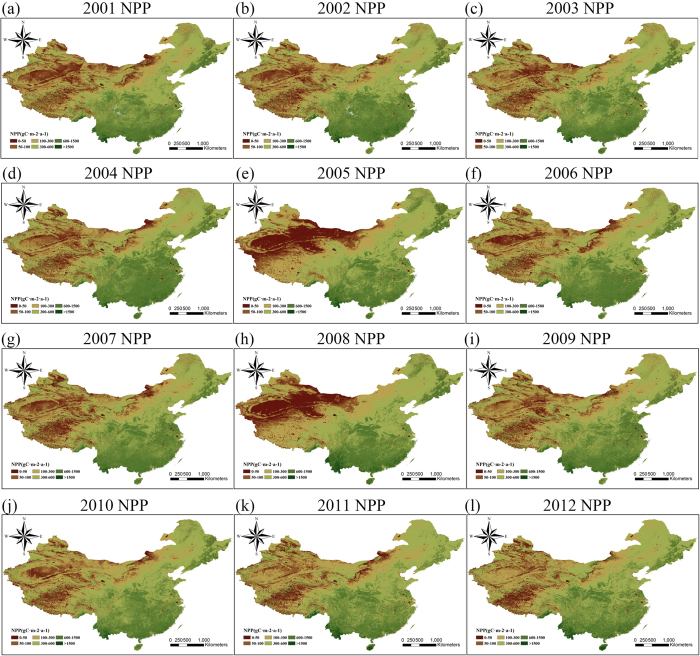
The evolution of NPP from 2001 to 2012. These figures were generated by ArcGIS for Desktop 10.0 (http://desktop.arcgis.com), ENVI v4.8/IDL v8.0 (http://www.esrichina.com.cn/softwareproduct/EI/ENVI/), and the MODIS Reprojection Tool (http://lpdaac.usgs.gov/tools/modis_reprojection_tool).

The NPP distribution from 2001 to 2012 in the study area, as simulated by the CASA model, is shown in Fig. 3, where it can be seen that the NPP presents a regular spatiotemporal distribution, and shows a decreasing trend from southeast to northwest.

The change trend was, however, different in different areas over the 12 years. The southeast of China showed only slight variation, while there was significant variation in the northwest area, especially in the eastern regions of Inner Mongolia and Xinjiang, which are marked in Fig. 1 as area 3 and area 1, respectively. We regressed the rate of variation of the 12 years of NPP values, obtaining the distribution of the change rate.

**Figure 4:**
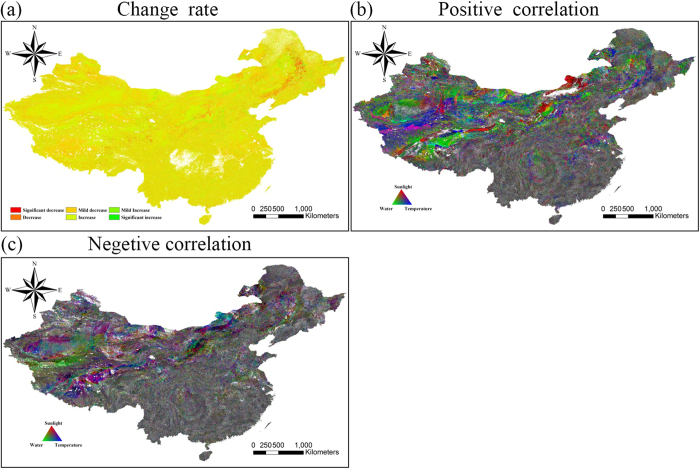
The NPP and meteorological factors over 12 years. These figures were generated by ArcGIS for Desktop 10.0 (http://desktop.arcgis.com) and ENVI v4.8/IDL v8.0 (http://www.esrichina.com.cn/softwareproduct/EI/ENVI/).

Figure 4(a) shows that the NPP showed a steadily increasing trend in almost all areas over the 12 years, particularly the eastern regions of Inner Mongolia (Fig. 1, area 3) and Xinjiang (Fig. 1, area 1). Nevertheless, the NPP showed a significant decreasing trend in the northeast region where the heavy industry cities of Harbin, Changchun, and Shenyang (Fig. 1, area 7) are located, as well as the Kunlun Mountains (the north of area 2 in Fig. 1). Because of the difference between different regions in NPP evolution, we calculated the correlation coefficients between NPP and the meteorological factors such as annual precipitation, annual accumulated temperature, and annual sunshine duration, to explore the main impact factors for the interannual variation of the different areas.

As Fig. 4 shows, we obtained two maps showing the distribution of the impact factors. The Fig. 4(b) represents a positive correlation between NPP and meteorological factors, and Fig. 4(c) represents a negative correlation. Every pixel denotes the impact of all three meteorological factors through additive color synthesis of the three prime colors of red, green, and blue. The red represents the contribution of annual sunshine duration to NPP, green represents the contribution of annual precipitation, and blue represents the contribution of annual accumulated temperature.

The three impact factors are evenly distributed in the central and south regions, but the NPP in the Huai River basin (Fig. 1, area 5) is positively correlated with annual sunshine duration and annual accumulated temperature, and negatively correlated with annual precipitation. For the northeast of China, the influences are even in the Daxing’an Mountains, the Xiaoxing’an Mountains, and the Changbai mountains (Fig. 1, area 6), while annual precipitation shows a positive correlation with NPP in the northeast plain. Both annual sunshine duration and annual accumulated temperature are positively correlated with NPP in the heavy industry areas such as Harbin, Changchun, and Shenyang (Fig. 1, area 7). For the Inner Mongolia steppe (Fig. 1, area 3), annual sunshine duration is positively correlated with NPP in the north-central region, while annual sunshine duration and annual accumulated temperature are negatively correlated with NPP in the central region. NPP shows a positive correlation with annual sunshine duration and annual accumulated temperature in the Kunlun Mountains (the north of area 2 in Fig. 1) and a negative correlation with annual precipitation.

**Figure 5:**
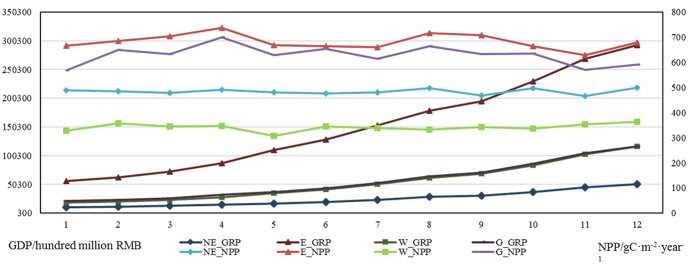
The NPP and GRP, with economic regionalization.

However, not only meteorological factors can influence NPP, but also human factors, through affecting the material circulation and energy flow of ecosystems and the land transformation caused by urbanization. We therefore divided the study area into four economic regions—eastern, middle, western, and northeastern—based on the divisions made by the 16th National Congress of China in 2002. The GRPs of the four regions show different trends depending on the different national strategy arrangements of the 11th Five Year Plan. Therefore, we compared the NPP pattern of the study area with the economic regionalization (Fig. 5).

As Fig. 5 shows, compared with the western and northeastern regions, the NPP of the central and eastern regions showed more significant fluctuations over the 12 years. The standard deviation of NPP for the central and eastern regions is 38.67 and 29.95, respectively, and the standard deviation of NPP for the western and northeastern regions is 14.87 and 11.46, respectively. For GRP, the eastern region showed the fastest rate of increase, whereas the northeastern region showed the slowest rate of increase. The western region and central region showed similar rates of change. We can see that there is a relationship between the economic growth rate and the fluctuation of NPP. The faster the rate of economic growth in a region, the more unstable its NPP, except for the central region, which showed the most significant fluctuations among the four economic regions, but only showed a low growth rate in GRP. To explore the different factors influencing the NPP, we used the three meteorological factors mentioned above, plus GRP and population, to calculate the correlation with NPP.

Table 1 shows the correlation between NPP and the impact factors, with economic regionalization. This shows that NPP in the eastern region is positively correlated with annual solar radiation at the 5% significance level and is positively correlated with annual solar radiation at the 10% significance level in the central region, indicating that solar radiation promotes NPP in these regions, while NPP in the two regions shows a negative correlation with the growth rate of GRP. There is no significant correlation between precipitation and NPP in the western region, while Fig. 4 shows that NPP in the northwestern region is positively correlated with precipitation. The reason for this is down to the difference in vegetation types in the northwestern and southwestern regions, which together comprise the western economic region. Other than the northwestern region with semi-dry grassland and dry desert, the northwestern region with humid and semi-humid forest^30^ makes the main contribution to the NPP of the western economic region, so that the correlation of the western region is different from the northwestern region. Because of the high latitude, the annual temperature is stable and low in the northeastern region, and has a negligible effect on the NPP of this region, which is also affected by pollution.

**Figure 6:**
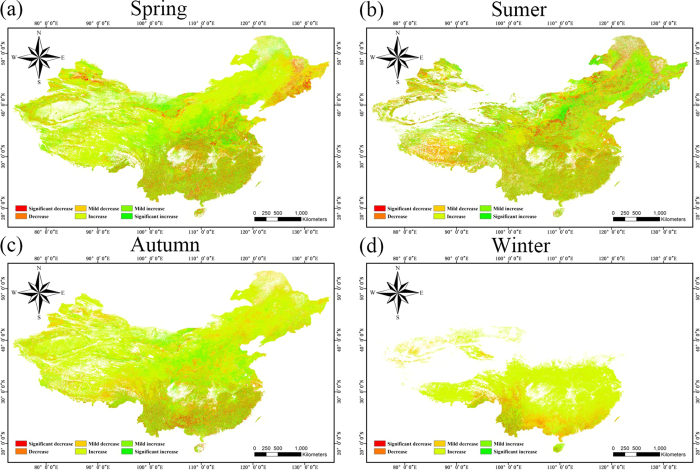
The seasonal change of NPP from 2001 to 2012. These figures were generated by ArcGIS for Desktop 10.0 (http://desktop.arcgis.com) and ENVI v4.8/IDL v8.0 (http://www.esrichina.com.cn/softwareproduct/EI/ENVI/).

To assess the seasonal change of NPP, we chose the NPP of March to May as the “spring” NPP, the NPP of June to August as the “summer” NPP, the NPP of September to November as the “autumn” NPP, and the NPP of December to February of the next year as the “winter” NPP. The final seasonal NPPs included 12 spring, summer, and autumn NPPs, respectively, and 11 winter NPPs, which was enough to show the different changes in NPP among the four seasons of the year. As Fig. 6 shows, the seasonal evolution of NPP is very different to the annual patterns depicted in Fig. 3. The NPP in the north of China has shown an increasing trend in spring over the 12 years, while the NPP in northeastern China (Fig. 1, area 6) has shown a decreasing trend. What is more, a clear boundary can be observed from the southwest to the northeast in the study area in Fig. 6(a). Because of the dry desert in Xinjiang (Fig. 1, area 1), the NPP is zero in summer as the desert becomes even drier. This is similar to the NPP in the north of China in winter under cold temperatures, which also shows missing data at this time of year. In Fig. 6(b), it can be found that there are several areas with obvious increasing or decreasing trends in summer. One area with a significant increasing trend is the northeast plain (Fig. 1, area 7), which has the highest latitude in the whole study area, alongside heavy industry. Another area with a decreasing trend in summer is the Tibetan Plateau (Fig. 1, area 2).

**Figure 7:**
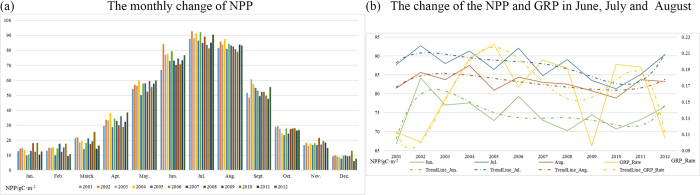
The monthly change of NPP from 2001 to 2012.

We also calculated the average monthly NPP of the study area over the 12 years, which effectively eliminates the invalid pixels. The results shown in Fig. 7(a) indicate that the trends are different in every month. The monthly average NPP always achieves the highest values in June, July, and August each year, with a slight fluctuation at the end of the year. To explore the correlations between human factors and NPP more accurately, we extracted the NPP of the three highest-value months and plotted this against the growth rate of GRP over the 12 years.

From Fig. 7(b), we can see that the NPP reaches a maximum in July, followed by August. Moreover, the NPP of the three months shows the same change trend over the 12 years, except for 2008. Based on the correlation between the growth rate of GRP and NPP, we can conclude that the increase of GRP has caused the decrease of NPP, which is consistent with the variation of NPP in June over the 12 years. In other words, the faster the economy develops, the lower the NPP in China, which can be considered as a warning that it is undesirable to develop the economy at the expense of the environment.

## Conclusion

In this study, we determined the monthly NPP of terrestrial China, except for the southwest of Taiwan and the South China Sea Islands, based on the CASA model and meteorological datasets for the period of 2001 to 2012, which includes the period of the 10th Five Year Plan made by the 9th National People’s Congress and the 11th Five Year Plan made by the 10th National People’s Congress. We verified the model NPP precision by the use of a ChinaFLUX dataset (eight sites), and the average relative error was less than 20% for five of the eight sites. The model NPP of all eight sites was within the range of the NPP of terrestrial China given by Zhu and Pan^29^. The distribution of annual NPP which we obtained presented obvious geographical characteristics, presenting a decreasing trend with the increase of latitude and longitude and significant interannual variation in the northwestern region. We obtained the rate of variation by regression of the 12 years of NPP values, and we explored the distribution by a spatial distribution analysis. The NPP showed low variability in almost all parts of the central and southern regions, a slight downward trend in the northeastern industrial region (Fig. 1, area 7) and Kunlun Mountains (the north of area 2 in Fig. 1), and a slight upward trend in Inner Mongolia (Fig. 1, area 3) and the Xinjiang region (Fig. 1, area 1). We also determined the distribution of the correlation between the model NPP and the different meteorological factors. The NPP showed different responses to the change of climate in the different regions, which means that the dominant factors were different. We also divided the study area into four economic regions based on the national development program given in the 10th Five Year Plan in 2001. We then calculated the mean NPP of each economic region and analyzed the effect of both meteorological and human factors. It was found that NPP in the eastern and central regions was significantly positively correlated with annual solar radiation, while NPP in these two regions was significantly negatively correlated with the growth rate of GRP. As NPP has shown a steadily increasing trend in almost all areas over the 12 years, we can see the great importance attributed to ecological issues by the Chinese government when developing the economy. For a more precise analysis, we also calculated the seasonal NPP over the 12 years, it and was found that there was a significant amount of invalid data in the winter and summer because the NPP is often zero in these seasons. The seasonal evolution of NPP was also very different to the annual pattern. In addition, we calculated the average monthly NPP of the study area over the 12 years, and it was found that the NPP always achieves the highest values in June, July, and August each year, so we used the data from these three months to analyze the relationship between the growth rate of GRP and NPP. This analysis suggested that the GRP growth rate has caused the NPP fluctuation, and the higher the GRP growth rate, the lower the NPP.

## Additional Information

**How to cite this article:** Wang, X. *et al*. Assessing the Spatiotemporal Variation and Impact Factors of Net Primary Productivity in China. *Sci. Rep.* **7**, 44415; doi: 10.1038/srep44415 (2017).

**Publisher's note:** Springer Nature remains neutral with regard to jurisdictional claims in published maps and institutional affiliations.

## Figures and Tables

**Table 1:** The correlation between NPP and the impact factors, with economic regionalization.

	Water	Sun	*t*	GRP	GRP rate	Pop.
Northeast	0.38	−0.22	−0.01	0.07	0.1	0.13
East	−0.28	0.57**	0.23	−0.34	−0.61**	−0.27
West	0.11	0.13	−0.08	0.38	0.1	0.39
Central	0.33	0.52*	0.43	−0.39	−0.56*	−0.37

We used an F-test to assess the joint significance of the factors. **and *indicate significance at the 5% and 10% levels, respectively.
